# A Smarter Health through the Internet of Surgical Things

**DOI:** 10.3390/s22124577

**Published:** 2022-06-17

**Authors:** Francesk Mulita, Georgios-Ioannis Verras, Christos-Nikolaos Anagnostopoulos, Konstantinos Kotis

**Affiliations:** 1 Intelligent Systems Lab, Department of Cultural Technology and Communication, University of the Aegean, 81100 Mytilene, Greece; canag@aegean.gr; 2 Department of Surgery, General University Hospital of Patras, 26504 Rio, Greece; georgiosverras@gmail.com

**Keywords:** smart health, IoT, surgical practice, Internet of Surgical Things

## Abstract

(1) Background: In the last few years, technological developments in the surgical field have been rapid and are continuously evolving. One of the most revolutionizing breakthroughs was the introduction of the IoT concept within surgical practice. Our systematic review aims to summarize the most important studies evaluating the IoT concept within surgical practice, focusing on Telesurgery and surgical Telementoring. (2) Methods: We conducted a systematic review of the current literature, focusing on the Internet of Surgical Things in Telesurgery and Telementoring. Forty-eight (48) studies were included in this review. As secondary research questions, we also included brief overviews of the use of IoT in image-guided surgery, and patient Telemonitoring, by systematically analyzing fourteen (14) and nineteen (19) studies, respectively. (3) Results: Data from 219 patients and 757 healthcare professionals were quantitively analyzed. Study designs were primarily observational or based on model development. Palpable advantages from the IoT incorporation mainly include less surgical hours, accessibility to high quality treatment, and safer and more effective surgical education. Despite the described technological advances, and proposed benefits of the systems presented, there are still identifiable gaps in the literature that need to be further explored in a systematic manner. (4) Conclusions: The use of the IoT concept within the surgery domain is a widely incorporated but less investigated concept. Advantages have become palpable over the past decade, yet further research is warranted.

## 1. Introduction

Advances in surgical practice have been rapid and non-stop for the past few centuries. From the groundbreaking idea by Joseph Lister that postoperative deaths might be attributed to certain invisible pathogens that could be combated with antiseptic solutions, to the popularization of robotic surgery in many modern medical centers, progress has been continuous. Many related advances came in the form of technological milestones that changed the shape of surgical practice as we know it today. The latest in a series of breakthroughs in the smart health domain is the utilization of the Internet in everyday surgical practice, the role of which is ever-expanding.

To systematically study the technological advances in a particular sector, attributed to the utilization of the Internet, the term “Internet of Things” (IoT) was introduced. While not strictly defined, IoT describes a network of Internet-based connected things equipped with (embedded) sensing and actuating devices, with data production, processing, and consumption abilities. The utilization of the Internet and IoT in medical practice can take many shapes and forms. Ranging from the awe-inspiring telesurgical procedures [1,2] to complex AI machine learning applications that aid in medical decision making [3], to a simple email containing a preoperative CT scan, the Internet of Surgical Things (IoST) is here to stay. A representative example of the IoST is a smart ingestible sensor (pill) that is activated after being swallowed [4], “travels” in the body through the colon and sends data to outer devices such as computers and smartphones when it detects a threat for cancer. Such a device can be used instead of colonoscopy for people who cannot obtain a colonoscopy due to psychological and physiological problems. In the broader aspect of the IoST context, IoST entities are different types of connected entities that “live” in this smart setting, such as surgical things (e.g., a connected surgery tool) [5], organs (e.g., a connected colon, an artificial heart) [6], humans (e.g., a connected patient or doctor) [7], smart devices [8] (e.g., a connected heartbeat monitoring device, a smart pill, an ingestible sensor), services [3] (e.g., a connected telemonitoring service), data (e.g., a connected data steam of heart monitoring data), etc. In this extensive survey, we aim to present an overview of current uses of IoT-embedded surgical practice by focusing on Telesurgery and Surgical Telementoring. As a secondary research question, we also briefly review the latest advances in IoT-associated image guided surgery, and surgical patient telemonitoring by utilizing the IoT paradigm.

Current literature on the Internet of Medical Things (IoMT) includes a multitude of heterogenous reports of Internet-based applications within the medical/healthcare domain. Most of the related articles present network(s) of sensor arrays and data processing stations, with or without actuating devices, interconnected via the Internet infrastructure. There is, however, a lack of a systematic approach within the existing literature as to how the IoT concept has revolutionized the surgical world towards smarter health. To fill this gap in current knowledge, the present systematic literature review studies the Internet of Surgical Things (IoST) domain by focusing on the three most prominent areas of application: (a) image-guided surgery, (b) telesurgery and telementoring in surgery, and (c) surgical patient monitoring [3,5]. Between these three areas of applications within the surgical discipline, the applications of Telesurgery and Surgical Telementoring are undoubtedly the most influenced by the IoMT concept. Therefore, our systematic review will be centered around studies of these applications. Additionally, we will briefly discuss the current literature on patient monitoring and IoT applications in image-guidance.

In this review, we systematically discuss a novel concept (the IoST) that is being rapidly incorporated into surgical practice. This is one of the first systematic review manuscripts covering this area. By focusing on the three applications described above, we provide a thorough understanding of the feasibility and effectiveness of different IoST applications. In addition, this paper also summarizes known and emerging weak points in IoST ecosystems that should be the focus of future research efforts. Finally, by incorporating data from system development studies, we offer insight into promising future uses of the IoST that are yet to be popularized but have the potential to be groundbreaking advances.

While there is a lack of a universally accepted definition for the Internet of Things, for the purposes of this review, we considered studies looking into ecosystems of interconnected computing devices, digital screens, sensor-bearing instruments, robotic surgery systems, mobile phones, 5th generation mobile networks, and can even include people (in our case either operators or patients. We have focused our attention on systems operating within the surgical world, connecting two or more “Things” (as defined above), via a certain, defined, and dedicated network. Looking into our specified research questions, some examples of IoT applications would be, for instance, a network comprising a preoperative imaging modality (e.g., MRI scanner), a processing station, and software within a specialized robot or specialized augmented reality glasses that ultimately aim to facilitate a procedure by superimposing real-time image guidance. Telementoring/Telesurgery systems usually comprise specialized working stations connected to a user that are capable of transmitting audiovisual cues and/or controlling a surgical robot at a distant location. Finally, telemonitoring is carried out by an interconnected series of sensor-bearing devices that centripetally transmit patient data, either directly to the physician, or to a dedicated data-gathering station.

The structure of the paper is as follows: Section 2 presents the research methodology, Section 3 presents an overview of the main results/findings of this survey, Section 4 discusses open issues and challenges, and, finally, Section 5 concludes the paper.

## 2. Materials and Methods

To meticulously look through the current literature concerning IoT in surgical practice, we have formed one primary and two secondary research questions that allow us to produce a thorough literature review. These research questions are as follows:

Primary:

1. What is the latest experience of telesurgery and surgical telementoring with regard to the IoT concept? (See Supplemental Figure S1).

Secondary:

1. What are the current applications of IoT technology in image-guided surgery? (See Supplemental Figure S2).

2. How can the IoT be utilized for patient monitoring outside the operating room? (See Supplemental Figure S3).

The presented literature review was conducted using the PubMed (Medline) and Web of Science (Clarivate) directories. The search queries for each research question as well as universal inclusion and exclusion criteria are presented in Table 1. We have used relevant keywords/phrases that characterize the articles of interest, for each of the research questions. To collect as many published articles as possible, we have incorporated additional articles from less specific queries, which can be viewed in Table 1. This was necessary mainly due to the lack of terms “Internet” or “Internet of Things” within the related keywords. Additionally, due to most of the IoT-related applications involving specialized sensor data inputs that lead to data-driven action, we have added a separate relevant query for all questions, thus capturing any surgical-specific system that would have been missed by the other search queries. The PRISMA flowchart of this additional search is presented in Supplemental Figure S4.

The presented review includes articles dating from 2010 to 2021. Through these years, the development of the Internet was constant, and, as a direct consequence, so was the incurrence of IoST-related publications. We have decided to include all relevant publications, even when reported methods seemed outdated for today’s standards (e.g., the use of dedicated land connections). By doing so, we have collected all reports of IoST-related concepts and showcased that the idea of an ecosystem of computing devices transferring data without the need for human interaction was present in the surgical sciences and practice long before high-speed or wireless Internet connections gave birth to IoT. Chronologically, we incorporated studies from 2010 onwards. This decision regarding the period was made largely to the fact that the IoT is a novel concept, mainly developed within the past decade.

Our search for related literature included articles published in peer-reviewed journals, as well as published presentations from conferences organized by notable scientific societies with a documented history of IoT expertise. We have included articles describing the clinical applications of the IoT concept specifically for surgical practice. Study designs that were considered for inclusion within the systematic review included clinical trials, case series, and animal trials. Several feasibility and prototype model studies were also incorporated if they presented clearly defined and well-supported potential for clinical application. 

The exclusion criteria of the survey concern the following article types: opinion or editorial articles, articles presenting a singular case report of previously a published methodology, and literature reviews of a subject. Such articles were deemed as low-quality articles that would not aid in forming a subjective overview of the IoST. We also decided to exclude articles that failed to indicate the presence of Internet-based interconnected devices. Articles that focused on technical developments rather than clinical applications (e.g., latency-lowering methods, advances in the security of IoT networks, advances in specific hardware or software components of a network, etc.) were also not included due to the clinically oriented nature of the review. Lastly, we excluded articles that were published in non-peer-reviewed platforms such as several online-only journals or pre-print platforms, due to concerns regarding their methodological quality.

The reference management tool EndNote was used for detecting duplicates. The PRISMA flowchart for each research question was developed, as can be seen in the corresponding Figures S1–S4. In the first step of the selection process, our research team excluded detected duplicate entries and articles that were clearly labeled as one of the study designs incorporated within the exclusion criteria. Screening of the records in the second step of the elimination process was carried out by revising available abstracts for any obvious signs of mismatching with the inclusion criteria (technically oriented studies, singular case reports). All studies deemed appropriate for further evaluation were sought for full-text retrieval. The fourth and final step of the review process included the evaluation of the full manuscripts, where the more specific exclusion criteria were applied (lack of clarity, unavailability of potential clinical use, etc.). This review process was the same for each of the three research questions. 

## 3. Results

Following a literature search and a gradual article elimination process, a total of 48 studies were selected for reviewing regarding our primary research question (Supplemental Figure S1). Of these studies, 36 were observational studies conducted within a clinical environment, evaluating either patients or healthcare professionals. Twelve studies were classified as feasibility or system development studies. In total, the studies included in this review incorporated data from 219 patients, and 757 healthcare professionals, and concern studies that use IoT in Telesurgery and Telementoring. The utility and main findings of each study is highlighted in the corresponding column of the supplemental tables. The studies in the field of IoST are rather few, and seemingly characterized by a high degree of heterogeneity regarding variables, study designs, and outcome types; therefore, it is not easy to identify a formal and systematic way to synthesize and report a cumulative and quantitative outcome. In that respect, in this paper we present a narrative review of the IoT in Telesurgery and Surgical Telementoring. 

Regarding our secondary research questions on Image-Guided Surgery (IGS), and surgical patient Telemonitoring, we systematically gathered 14 and 19 studies, respectively. Studies regarding the IoT in IGS incorporated experimental data from 111 patients and 35 patient models in total. Of the included studies, 7 were clinically based, and another seven were system development/feasibility studies. As for the IoT in surgical patient Telemonitoring, of the 19 studies, 15 were clinical studies, and 4 discussed newly developed IoT-based telemonitoring systems. 

### 3.1. The Internet of Telesurgery and Surgical Telementoring

**Figure 1:**
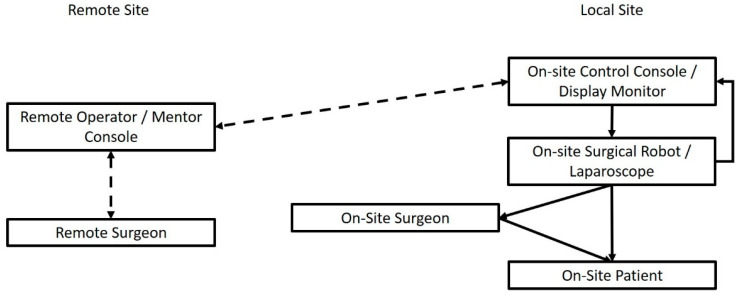
Connected IoST entities and workflow of an IoST-based Telementoring/Telesurgery System.

Perhaps the most impressive advancement the technological applications were able to provide in the surgical world is the incurrence of the telesurgical procedures. When discussing the prospects of IoT applications within surgical theaters, there are two key concepts in existence to keep in mind: telesurgery, and telementoring. While telesurgery describes the performance of a surgical operation by a remotely located surgeon, utilizing a network of devices with the aim of transferring the surgeon’s haptic commands to a robotic surgery system, telementoring is the performance of live surgery on-site, with the live assistance of a more experienced surgeon, located off-site [9,10,11,12,13,14,15,16,17,18,19,20,21,22,23,24,25]. In order to achieve the latter, a network of connected cameras, microphones, screens and computers is necessary [25,26,27,28,29,30,31]. A fundamental outline of the network and crosstalk required is depicted in Figure 1. This connection is mostly provided through the Internet nowadays, and with new and emerging technologies, such as the fifth generation of mobile networks, implementation of teleservices is expected to grow (Supplemental Table S2). A 2017 systematic review of the literature on telementoring in the operating room revealed that operating times and complication rates were the same when compared to on-site live mentoring of younger surgeons [32]. This showcases that telementoring possibly has the potential to supplement live surgical training and might allow trainees to even surpass their educational goals when mentored remotely.

In addition, telementoring has the potential of long-distance consultation with senior and specialized surgeons who can provide live assistance in the operation rather than a simple preoperative consultation, or the costly option of transferring difficult to manage cases in specialized units [15,16,17,18,19,20]. Systems that are developed for surgical telementoring in live surgery can also be expanded with an array of biosensors for physiological parameter monitoring that is transmitted directly to the mentoring surgeon [27], allowing for the complete monitoring of the surgical patient, rather than just the surgical procedure. Studies evaluating the satisfaction outcomes of surgical trainees whenever live proctoring from a distance was used reveal universally high satisfaction rates [18,19,20,21,22,23,24,25] that were common to both mentors and mentees. Participants of said studies felt that telementorship sessions with more experienced surgeons would be a useful addition in surgical education curriculums [33,34,35,36,37,38,39,40]. Even when utilized for teleconferencing and simple audiovisual transmission of an operation for teaching purposes, participants rated positively to their experience more than 90% of the time [33,41,42,43]. This goes to show that the introduction of the Internet in the everyday surgical practice can have a pragmatic impact in the education of younger surgeons, particularly at low costs and without complex institutional requirements. Telementoring in surgery allows the safe transfer of knowledge and skill aptitude from a more experienced surgeon to a younger mentee.

Studies reporting telementoring techniques within the OR were split broadly in the two categories of the studied teaching method. The simplest form of surgical telementoring includes the live observation of a surgical procedure by a distant senior surgeon who can provide audiovisual guidance through an Internet connection, or a more advanced form of proctoring including the distant manipulation of a camera-bearing surgical robotic arm.

A study by Hinata et al. [28] compared in a systematic manner the perceived differences in live mentoring with telementoring in robotic surgery. Apart from requiring a stable and fast internet connection being the single drawback, all of the postoperative parameters of the surgical patients were the same between the differently mentored groups in almost all the included studies. In another comparative study of internet-based telementoring versus in-person telementoring, not only were the postoperative results the same between the two groups of mentees, but the mentors also showed a significant trend towards telementoring [27], while in some studies, mentees achieved better postoperative results when distance-mentored [43,44,45,46,47,48]. In a report on telementored trainees that performed bariatric surgery procedures, those that utilized Internet-based mentoring achieved fewer complications, shorter operative hours, as well as shorter hospitalization time [1]. In addition, Altieri et al. studied whether delivering a surgical skills course over the Internet would be less effective than live mentorship [29]. After the course was completed, the trainees were also found not to perform similarly in the post course assessment, but also showed non-inferior skill decline patterns for a few weeks after the course. These results prove that internet-based mentoring in surgery can be similarly effective in the long-term skill-honing process and is not confined in short-term positive outcomes. Additionally, they found that within every study describing an internet-based telementoring system, there were almost no unexpected intra-operative complications [47]. This proves that the use of the Internet as a mentoring tool can be a safe alternative, even when within a high-risk environment such as the operating room (OR).

With the incurrence of high-speed network connections, the handling of larger data transmissions at no expense of the perceived live mentoring was made possible. Utilizing such connections, several research teams were able to integrate AR within surgical telementoring. In these publications, authors describe the use of specialized AR glasses by the mentees [10,33,34,35,36,43,44,45,46,47,48,49,50]. The glasses were equipped with cameras providing live feeds of the surgical field that were transmitted through the Internet to a distant mentor. The mentoring station on the other end includes motion tracking sensors and cameras that capture the mentor’s movements. These movements are superimposed on the mentee’s field of view and on to the surgical field through the specialized glasses in order for the mentee to closely follow along. The trainees assigned to be mentored with this system achieved higher scores on operational evaluation and were prone to less mistakes [10,36,43,44,45,46,49,50,51,52,53]. In one clinical study, AR telementoring was compared with simple telementoring through audiovisual transmission and audio guidance [44,48]. People mentored with the AR system made less mistakes and were more accurate. However, in several similar studies, authors noted that mentees on such systems required more time to complete the simulated procedure [32,34,43,44]. Additionally, we must not forget the increased cost of such systems, as they require not only high-bandwidth internet connections, but sophisticated equipment for the mentor and mentee stations. Such equipment may not be readily available at most institutions. Andersen et al. [10] took the AR systems a step further by incorporating pre-recorded footage of similar operations that could be viewed directly onto the mentee’s display. Operative results were encouraging; however, the individual variability in patients posed a drawback, since the pre-recorded operations could not account for variability.

More advanced telementoring systems include the ability of the remote mentor to assume control of part of the operating system, most commonly the laparoscope-containing arm of the surgical robot [40,42,45,47,48]. Advantages of this setup is the ability to provide direct feedback to the mentee and demonstrate the appropriate handling of the instruments, even though the mentor is located remotely. In our group of studies evaluating similar setups, all reported operations were carried out successfully by the mentored surgeons without intra-operative complications. Mentees also reported that they preferred the distant mentoring to on-site mentoring. In a study by Prince et al. on simulated surgical tasks [14], mentored surgeons that utilized the telementoring system who allowed for instrument control by the mentor scored higher on dexterity assessment tests than those who did not.

Telementoring with the use of Internet connections in surgical specialties, however, is not limited to the operating room. Authors have reported utilizing telementorship to organize skill stations, assess the work of mentee surgeons, broadcast live operations for teaching purposes, teach postgraduate courses, conduct virtual grand rounds, etc. [33,34,35,36,37,38,39,40,41,50,51,52,53]. In all of these activities, the mentees rated their experience almost universally positively. A recent endeavor by Greenberg et al. [39] evaluated a novel surgical skill simulation course that incorporated AR. Post-course evaluation revealed not only satisfied students, but increased aptitude as well. Suzuki et al., developed a surgical training system, using software that could provide an accurate virtual surgical case and robotic controllers that could be connected to the mentee’s PC for a training session. In addition, the software, in addition to the patient models, was available as a cloud-based product, meaning that the exact same simulation can be accessed from anywhere in the world.

Indeed, distance mentoring with the use of the Internet also has certain drawbacks. Firstly, as it is often underlined by authors, all the systems described here require a stable, and, in many instances, high-bandwidth Internet connections. This is often an issue with smaller institutions, or institutions at developing countries. In addition, many of the systems included here, require much more than a webcam and a microphone. Complex setups for surgical telementoring often include robotic systems, expensive software, AR glasses, specialized simulation instruments and more. All the above can prove to be a substantial cost that reaches prohibitive status in certain healthcare systems. Trainees that make use of telementoring systems outside the operating room, have also reported that the experience, although positive, could never replace their presence within the OR [39]. In several studies, authors report one or more instance of technical difficulties with the remote mentoring systems, such as audio or video failure, latency times or interruption of connections [42]. Seeing that these studies are mostly dated prior to 2015, we can hypothesize that such difficulties were a result of inferior internet capabilities, an issue largely resolved in today’s practice, where fast internet speeds are widely available. Scheduling of the mentoring sessions can also prove to be a minor issue when the mentor and mentee are in different time zones. However, this is not something that careful planning cannot address. Lastly, there is also an additional hindrance mentioned by authors in telementoring and telesurgery. This is none other than legal considerations that must be addressed. Although not expected to differ significantly than standard practice, there is still a gap of specific legislation around telemedicine in general that will need to be filled before telemedicine services are offered as a standard of care.

Telesurgery is closely related to another relatively recent advancement in the operating room: robotic surgery. In fact, robotic surgery (the next logical step to laparoscopic surgery) was crucial in planting the idea that if a surgeon can command a machine and perform a procedure from a few meters away, why not apply the same principle in larger distances, perhaps even transatlantic ones. In theory, by utilizing a complex internetworked system of cameras, video streaming, feedback data and data processing and transmission devices, the remotely situated surgeon is able to operate the robotic surgery system and provide real-life, quality surgical outcomes. In this scenario, the IoT concept is mainly applied in the collection, transmission and exploration of the collected data, as well as within the bidirectional transmission of signals. The data processed and transmitted include audio, video, and images. In some experimental systems, where remote control of robotic surgery modalities is involved, the data also include feedback related to the positioning of the robotic arms, the surgeon’s hand positioning and movement. The major impending factor in achieving this, however, was none other than the delay, caused by relaying large amounts of data in a wireless manner over large distances. The first recorded instance of a long-distance surgical procedure was an experimental cholecystectomy performed by the team of Marescaux et al. on swine specimens [40]. In their experimental study, all operations were successful, and the measured latency between the command given by the remotely located surgeon, and the observed response was 155 ms, indicating future perspectives where such surgeries might be part of the everyday practice. The first validation that performing surgical procedures from a distance was feasible and safe came in 2002 from the same research team [47] that performed the first ever cholecystectomy on a real patient, utilizing a robot that was controlled overseas.

Since then, the telesurgery concept has come a long way, with one major milestone being added recently, namely, the incorporation of 5th generation mobile networks in surgical practice. Study groups evaluating the feasibility of remote-controlled robotic surgery systems have found that it is a realistic option for performing surgical operations, with no additional risk for the patient and comparable surgical outcomes [42,43,44,45,46,47,48,49,50,51,52,53]. The feasibility of remote surgery application is such that surgical teams have achieved successful operations with optimal postoperative results from a remote site of more than 3000 km away from the primary site where the operating suite is located. This means that patients are now able to receive specialized surgery without having to relocate to a specialized surgical center. Utilization of high-speed internet connections is usually in the form of fiber optic connections, or more recently 5G mobile networks, and allow minimal latency within surgical practice, meaning increased safety for the patient as the operator is confident of their movements within the surgical field [39]. Within the included studies, all of the authors report no intra-operative complications, in addition to no further time delays of the surgery. Therefore, we can only expect for direct telesurgery with remote handling of surgical robots to be further expanded in the future, making surgical care available for less developed areas in a safe and highly efficient manner, comparable to live surgery. However, before widespread implementation, key issues of telesurgical systems need to be addressed [41,42,43,44,45,46,47,48,49,50]. These include further limitation of latency between stations, addressing safety concerns regarding cyber security, as well as the newfound liability due to medical damage caused by remotely operated robotic surgery systems. Financial imbalances between populations and countries are perhaps the greatest obstacle to the popularization of long-distance surgery. However, as the years progress, the availability of stable and fast Internet connections is on the rise and the costs of medical infrastructure, while substantial, can be surpassed by the cost-effectiveness of such advances [48].

Authors have also compared the different options of Internet connections that were applied to telesurgical procedures, namely, land cable connection with satellite connection. No differences in operative parameters such as blood loss or total operational time were found, and the participants felt equally confident with both modalities. In one study, the satellite Internet connection produced significantly greater latency times between the operator and the surgical robot; however, they were not sufficient to constitute the operation as unsafe.

### 3.2. Image-Guided Surgery in the IoT Era

The basic principle of Image-Guided Surgery (IGS) is constituted by the utilization of a tracking device, alongside pre or even intra-operative imaging, to aid the surgeon in the spatial orientation during a surgical process. (Supplemental Table S1). Authors of relevant publications have used IoT networks to incorporate patient imaging, as well as preoperative planning, into the operating room.

**Figure 2:**
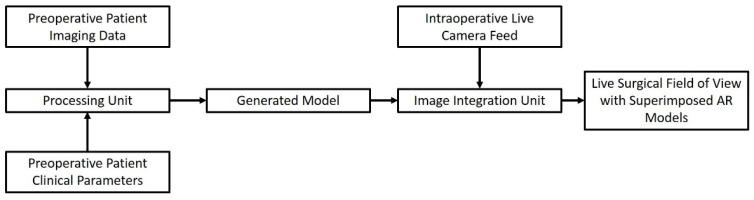
Connected IoST entities and workflow of an IoST-based Image-Guided Surgical System.

The network-based sharing of data and the creation of a workflow through sequential data appraisals and data provision towards the next component makes IGS a prime example of an IoT application in surgery. An IGS system built around the IoT approach usually consists of input of preoperative imaging data of the surgical patient. These data are then used to make reconstructed models of the anatomical area of interest. Such models are then transmitted wirelessly to a modality of choice, ranging from augmented reality (AR) glasses to the viewing screen of a surgical robot [54,55,56,57,58,59]. A simplified schematic illustrating the workflow of these systems can be seen in Figure 2, encompassing the idea of the IoT concept defined as a network of inter-connected devices that process and exchange data.

Current literature in IGS is heavily referred to as advances in the neurosurgical field. In this aspect, Internet-based IGS has a lot to offer by incorporating data from various sources and making them available to the surgeon in real-time. Augmented reality systems make use of the Internet to transmit preoperative renderings and patient imaging after registering these data with on-site patient images onto specialized smart glasses [54,55,60]. Coupling preoperative patient imaging with the image-guided system usually involves using certain sensors for the tracking of specific markers within the patient [55]. Studies on real-time surgery indicate that IGS has been very helpful in the identification and preservation of vital structures [58,59,61]. Almost all of the reports included here report that AR imaging systems allowed the preservation of vital structures in real-life patients [60,61,62,63,64], significant accuracy when utilized to assist in biopsies or electrode insertion [54,55,56,57,58,59] and real-time compensation for brain shift during neurological surgery [54], a novelty not achievable otherwise. In a study by Watanabe et al. [60], the intra-operative tablet devices, used to provide live camera views of the surgical field were also tracked in space by an overhead multi-angle camera system and special tracking spheres [60,61,62,63,64]. Internet use within the surgical suit enabled authors to utilize pre- and intra- operative patient imaging to create a live overlay of anatomical areas of interest (such as tumors or sensitive nearby structures) by connecting the preoperative image repository with an image processing software that returned the final image data either to a monitor or another specialized apparatus (e.g., smart glasses) [59].

Eftekhar et al. [63] took the AR integration within the surgical suite a step further by introducing a lesion-tracking smartphone app for mobile phones. The software will utilize the smartphone’s camera to register anatomic landmarks of the patent’s head surface that will be used to align the postoperative imaging with the live image. Therefore, we can see that the Internet can be used, not only as a “data highway” for short-term data handling, but as a connection to large servers, allowing direct access to a repository of patient data.

Related work by Guo et al. [8] and Li et al. [54] describe the integration of surgical robots within IGS systems. The coupling between the intra-operative MRI imaging, the surgical robot, the central processing unit and the end-display of the processed imaging data is achieved by utilizing wired or wireless Internet connections. Despite lacking trials on their performance in real-life surgery in humans, it is safe to say that the Internet in this case, has provided reliable interconnectivity with multiple appliances at once, and has the potential to become the unseen substrate of modern neurosurgical breakthroughs.

Ushimaru et al. [5] utilized RFID tracking tags placed on laparoscopic instruments in order to track usage in general, as well as the activation times of each tool. Data from the tags were transmitted onto RFID readers within the operating room. The RFID readers then transmitted the recorded data to specialized computer software that converted the electrical current readings to “on/off” indications and activation times. This experimental setup was successful in capturing the usage patterns of surgical instruments during cholecystectomy. This short proof of concept study opens the door towards incorporating network-based tracking systems within the everyday surgical practice that will capture surgical instrument use and possibly aid the surgeon in spatial navigation. Possible implications of this could include coupling a similar tracking system with an AI network that can aid in intra-operative, real-time decision making, or an in-hospital data accumulation system that will be able to measure surgical performance. A research group was recently able to combine the tracking of virtual laparoscopic instruments operated by a distant mentoring surgeon with the live feed image of the laparoscope and the real surgical instruments operated by a mentee [58]. Meier-Hein et al. also managed to construct a tracking algorithm for laparoscopic instruments by using sensor data and an Internet-based integration status. Their system was successful in producing an algorithm that could automatically detect and accurately annotate the laparoscopic surgical instruments, as well as their usage status in real-time operations.

On the other hand, the cost of implementation of such technological advances might prove to be restricting for certain institutions [54,55,56,57,58]. One of the biggest drawbacks of the systems described in the literature is that the vast majority of them report small patient cohorts and lack a direct comparison with other, more traditional surgical methods. Some authors even reported comparable accuracy when they compared novel IGS systems with their simpler, older counterparts [59]. Other reports that describe exciting new advances in the field of IGS, with promising results in simulations of surgical operations, lack a patient application altogether [8,60]. In that aspect, the precise effect the Internet has had on the evolution of IGS is harder to quantify. Still, we cannot deny that advances in the connectivity of devices achieved by an internet connection is a major component towards the modernization of medicine services in general, and surgery in particular.

### 3.3. The Role of the IoT in Telemonitoring the Surgical Patient

**Figure 3:**
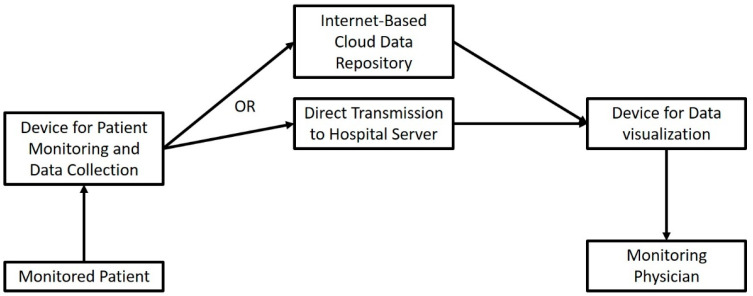
Connected IoST entities and workflow of an IoST-based Telemonitoring System.

In order to complete our appraisal of the IoT concept in surgical specialties, we would be remiss if we did not include the potential uses outside the operating room (Supplemental Table S3). Medical telemonitoring usually consists of a specialized “smart” device that captures target parameters and transmits them through a wireless Internet connection, either directly to the referring physician, or to a centralized repository from which they can be accessed (Figure 3). The role of the Internet here is more straightforward: instead of being the network substrate that interconnects a variety of operating stations, data repositories and data processing modalities, here, it is used as a unidirectional “data highway” that runs towards the physician. In contrast to previous advances, telemonitoring has been widely implemented in some healthcare systems.

Within the surgical patient subgroup, there have been a few clinical studies looking into the applicability of telemonitoring, usually in the postoperative period [3,65,66,67,68,69,70,71,72,73,74,75,76,77,78,79,80] (Supplemental Table S3). Patients enrolled in an at-home monitoring program after chest wall surgery were also monitored effectively by utilizing the Internet to input certain parameters in an online platform [65,67] Cardiac surgery patients were also studied in an IoST rehabilitation program that included wearable biomedical and motion tracking sensors [68]. The physician was therefore able to monitor the patients’ activity levels and their performance in rehabilitation exercises. In a 2021 study by Cos et al., patients scheduled to undergo pancreatic surgery were monitored preoperatively by using a wearable smart device that was able to record heart rate, activity status, etc., and through an internet connection, transmit them to a central server. Not only did patients adhere to this novel concept, but the data that were automatically collected were of such quality that the research team developed an accurate predictive model for postoperative outcomes [3]. Biosensor-based systems are able to wirelessly transmit data on physiological parameters of the patients in order to assist with postoperative monitoring. Authors have reported the incorporation of pulse rate, blood pressure and activity tracking sensors as being successful in monitoring the rehabilitation process of surgical patients [69,70,71,72,73,74,75,76,77]. Reported advantages, include successful vital signs readings, short training period of nurses and patients alike, less unplanned office visits, and predicting unplanned postoperative complications by indirect monitoring of vital signs [8]. Kim et al. successfully developed a Doppler cuff that could be remotely monitored, allowing remote monitoring of the blood flow of skin flaps. This resulted in superior graft survivability rates [6]. Results such as these are indicative that we have come to a point at which smart devices with Internet connections can provide fast and accurate measurements of clinical parameters in a reproducible manner and have the potential to effectively substitute an in-office visit for routine monitoring. In addition to the universally observed accuracy of the requested parameter measurements, distance monitoring saves time for the patient and the physician alike, prevents missed appointments and is generally preferred by patients [78,79,80]. When studying the response of bariatric patients postoperatively, regarding the telemonitoring process, Vilallonga et al. found that patients themselves would prefer the telemonitoring option [71].

Postoperative monitoring of physiological parameters with the use of biosensors can be further expanded to include automated action after the received sensor signal. In an example by Wang et al. [75], patient-controlled analgesia was administered after indications from a biosensor feedback system that measured physiological responses to pain. Postoperative pain and nausea were reduced in the patients treated with this system. An ostomy alert sensor was developed by the team of Rouholiman et al. [80] that was capable of alerting nurses, patients, and physicians alike of the content status of the ostomy.

The vast array of remote monitoring systems described in the literature could very well be applied to a pre- or post-operative surgical patient, even if there are currently no studies for this specific cohort of patients. Systems used to monitor diseases such as heart failure, hypertension, pregnancy-related complications and more can easily be applied to the surgical patient in the future. A recently developed smartphone application could aid in the distant monitoring of COPD patients and could be useful in the detection of acute exacerbations and advise timely hospitalization [74]. All the above-mentioned modalities for long distance patient monitoring rely on the Internet for data transmission and could very well see their way in surgical patient monitoring or consultation in the not-so-distant future.

There are of course some hurdles still left in the way of universal internet-based patient monitoring. The biggest of which seems to be the reported difficulties elderly patients have with operating such systems [74,75,76,77,78]. Technological illiteracy is a persistent issue that seems harder to address than the technicalities of the systems. Patients of older age, of mental burden and patients without a reliable Internet connection that is readily accessible are in danger of being left out of such technological advancements, an observation reported in the majority of clinical studies. What is more, home-based distance monitoring relies entirely upon the adherence of the patient in data recording and the use of the instructed devices.

## 4. Discussing Open Issues and Challenges

The present literature review showcases current uses of the IoT paradigm within surgical practice, mainly by exploring the concept of telesurgery and surgical telementoring.

Telesurgery and surgical telementoring are undoubtedly the most impressive of the listed IoT applications within the surgical practice. Looking into the included particles of the presented survey, we can safely say that long-distance surgery on real-life patients is now feasible, although it seems to be scarcely performed. Authors do not mention any surgical safety compromises when IoT networks were utilized to perform telesurgery, which is most definitely the first hurdle that a newly emerging technique must overcome on its way to popularization. However, the most significant drawback of these applications, is the requirement of fast, stable Internet connections that will allow minimal latency and data loss. Paradoxically, such Internet connections may be lacking in the areas that are most in need of telesurgical and telementoring applications, such as rural and distant institutions that are not able to provide expert surgical consultations. Popularization of 5th generation (5G) mobile Internet networks is expected to be a big step towards that direction that will guarantee minimal latency and maximal connection stability. Particularly for telesurgical applications, there is also the issue of legal implications, as is underlined by several authors. The lack of specific legislature regarding long-distance surgery might prove to be grounds for liability of the providers, which is not previously described or covered by insurance. Due to the rapid implementation of such systems that is expected to follow, we must tackle such issues rapidly so that providers feel confident in partaking in long-distance operations or consultations. Financial costs are once again a key factor in play here. Teleconsultation or telementoring might not require much more than an audiovisual connection over the Internet; however, telesurgery itself requires surgical robots in order to transfer the instrument movements as instructed by the surgeon. Once again, these are not available everywhere on the globe, and rural centers in need of distant expert consultation are not likely to have robotic surgical systems available. From the above, it can be concluded that the feasibility and reliability of a new paradigm such as the IoST in telesurgery are not guarantees for its widespread application. There are still major logistic problems to overcome before telesurgery becomes part of the everyday surgical practice. Our research team aims to set up the first surgical telementoring system in Greece that will begin by providing a real-time audiovisual connection between an expert and a novice surgeon with live intraoperative guidance. This system will be evaluated against the traditional live mentoring of more inexperienced surgeons in order to provide a proof-of-concept. Further steps within our goals also include the introduction of more surgical centers to the said system in view of establishing a network of interconnected hospitals that provide regular surgical consultation over the Internet.

Image-guided surgery is one of the most developed forms of operative strategy due to the minimization of tissue damage, blood loss, operative hours and postoperative pain. By making use of serial interconnected data processing modules, researchers are able to construct IoT networks that greatly facilitate image-guidance in surgery. The real-time integration of preoperative imaging is the main goal in this case. The tracking of the imaging-specified patient anatomy in real-time surgical operations has proved highly significant in increasing surgical accuracy, and, in many cases, in assisting the prevention of accidental tissue damage. Despite encouraging results from proof-of-concept studies, however, these systems seem to be lacking adequate investigation in large-scale patient cohorts. As such, it is not safe to state with confidence that image-guided surgery incorporating the IoT paradigm today is widely accepted for clinical use. Still, we can safely conclude that IoT-based tool-tracking sensors will be proved valuable in the near future, especially for the surgeons that require maximum precision in instrument or anatomical landmark tracking. A major advantage of these systems, as already mentioned by a large number of authors, is the capability of IoST to account for interpersonal variability in anatomical structures in a real-time manner. Despite the lack of explicit reference in any of the included studies, acquisition of the necessary tool-tracking sensors and software is surely expected to be a major issue for several institutions. Therefore, the scientific community ought to aim for larger studies on surgical patients that will not only include image-guidance systems similar to those mentioned here but will randomize patients between image-guidance systems in order to better delineate the proposed advantages over older systems.

Long-distance monitoring using IoT is also one of the most common applications in medicine and healthcare. There is a limited number of articles including telemonitoring of the surgical patient specifically; however, such systems have proven their value in patient comfort and effective physiological parameter monitoring. The major challenge in the widespread implementation of said systems is the technological literacy of the patient population, as well as its Internet access. While improving Internet access is an ongoing global strife, researchers must focus on constructing adaptable and more intuitive user interfaces of such applications in order to appeal to older patients not comfortable with the everyday use of technological applications, wearable sensors and smart devices. Patient adherence is a challenge within these studies. The popularization of long-distance monitoring, in addition to patient education for these systems, will assist with the wider adoption of IoT-based monitoring of the surgical patient.

## 5. Conclusions

The aim of the present literature review is to collect and analyze the available knowledge on the most prominent fields that the IoT paradigm finds application to the surgical practice, i.e., the Internet of Surgical Things. Technological advances allow the incorporation of rendered preoperative data to the live surgical field, the valuable from-a-distance mentorship of younger from more experienced surgeons, the realization of a surgical procedure by remotely controlled robots, and the monitoring of surgical patients without the need for hospital visits. Despite the availability of reliable and fast Internet being a requirement for the actualization of these concepts in more areas of the world, the seamless incorporation of “smart” functions within the surgical world with the aid of the Internet is on a steady route to becoming a reality towards smarter and more efficient health services.

This study faces certain methodological limitations which arise from the narrative structure of most of the included literature. To begin with, this is a narrative systematic review, meaning that there are no statistical deductions to be made or pre-specified comparisons between different methodological approaches. The IoST is a concept, rather than a method, and therefore tangible comparisons can only be made in very specific applications. Current literature on IoST is lacking in comparative studies that produce results one can use to reach safe conclusions. Despite adhering to specific selection criteria, our study selection process was amenable to an unavoidable degree of bias, arising mainly from the lack of specific clinical applications in many of the screened publications. Finally, our inclusion of feasibility and animal model studies needs to be interpreted as a showcase of potential future applications, rather than everyday uses of the IoST concept. The review’s findings can be summarized, per the research question, in the following paragraphs.

Telesurgical applications are undoubtedly the primary example of IoST systems. Their validity has been repeatedly evaluated over the years and the literature reveals that telesurgical networks are routinely incorporated in many surgical centers. Within such systems, there is a wide adoption of sensorial arrays that transmit data to distant locations. Telementoring is an even more inclusive concept in surgical education. Evaluation of distant teaching in surgery has revealed that it is a viable alternative to traditional teaching, which is at times preferred over in-person assistance. Recent literature also points out the incorporation of 5th generation cellular networks that are able to effectively eradicate latency times and connectivity issues.

IoST in image-guided surgery is currently an “under development” application that has produced tangible results in only a few clinical studies. Intraoperative use of IoST networks mainly focuses on superimposing preoperative imaging on live surgical camera feeds to assist surgeons in precision-requiring tasks. Despite being investigated mainly in neurosurgical procedures, these networks are predominantly software-dependent, thus making it possible to also be incorporated in more procedures in the years to come.

Patient telemonitoring involves the utilization of network-connected biosensors that track physiological patient parameters that are observed by a distant physician. Such sensorial ecosystems seem to be highly successful at detecting specified cutoff points and providing alerts to the physicians. Additionally, such systems can shorten hospital stays and lessen routine in-office patient visits, without compromising patient safety. Lastly, telemonitoring has relatively few requirements, considering the availability of biosensors in everyday technological products and “smart” wearable devices.

## Figures and Tables

**Table 1:** Search Queries.

Research Question	Queries Used in Medline	Queries Used in Web of Science
What are the current applications of IoT technology in image-guided surgery?	(image-guided surgery) and ((internet of things) or (internet))	(ALL = (image guided surgery) and (ALL = (internet of things) or ALL = (internet)))
What is the latest experience of telesurgery and surgical telementoring with regard to the IoT concept?	(telesurgery) and ((internet of things) or (internet)) + (telementoring) and (surgery) + (telesurgery)	(ALL = (telesurgery)) and ALL = (internet) + (ALL = (telementoring)) and ALL = (surgery) + ALL = (telesurgery)
How can the IoT network be utilized for patient monitoring outside the operating room?	((telemonitoring) and (surgery)) and (internet) + (surgery) and (internet of things)	((ALL = (telemonitoring)) and ALL = (surgery)) and ALL = (internet)
Supplemental Query for all Research Questions	(sensors) and (surgery) and ((internet) or (internet of things))	((ALL = (sensors)) and ALL = (surgery) and (ALL = (internet) or ALL = (Internet of Things)))
Inclusion and Exclusion Criteria		
Inclusion Criteria	Articles that described clinical or feasibility studies of modalities incorporating the IoT framework. Articles that described a system in development, were able to demonstrate potential real-life application.	
Exclusion Criteria	Case reports of previously known modalities. Literature reviews. Opinion or Editorial articles. Lack of clarity regarding the utilization of an internet-based network. Articles focusing on technical developments rather than surgery-oriented application potential. Unavailability of text. Articles solely in non-English languages. Articles from pre-print servers or online-only publication platforms.	

## Data Availability

Not applicable.

## Supplementary Materials

The following supporting information can be downloaded at: https://www.mdpi.com/article/10.3390/s22124577/s1. PRISMA methodology (Supplemental Figures S1–S4), Supplemental Tables S1–S3.
