# Supplementary material for: A Smarter Health through the Internet of Surgical Things
Source: Sensors (Basel). 2022 Jun 17;22(12):4577. doi: 10.3390/s22124577 (PMC9231158; doi:10.3390/s22124577)
Supplement: Supplementary file 1 [file sensors-22-04577-s001.zip › sensors-1689180-supplementary/Supplemental Table S1.pdf]

Supplemental Table S1: Studies evaluating the IoT in Telesurgery or Surgical Telementoring

Studies Evaluating the IoT in Telesurgery or Surgical Telementoring	Type of Study	Evaluated approaches – Connected IoT entities	No of Participants	Proposed Advantages	Limitations
Patel et al (2021)[15]	Study on Trainees	Surgical Training Tasks with the aid of webcam-delivered mentorship.– Connected Mentors, Mentees and Presentation Tools	21 Students	The system was easily understood by students. High satisfaction scores when grading the teaching system. No problems with audio/video feed.	The mentoring laptop and the robotic system's screen meant that the participant had to avert their gaze. Some instances of video quality temporarily dropping.
Morohasi et al (2021)[47]	Simulation of Surgical Tasks	Evaluation of the connection between 2 hospitals in a distance of 150km was tested.– Connected Surgeon to Surgical Robot	12 Surgeons	The recorded delays were minimal, and were not expected to have any impact on real-life scenarios.	-
Chu et al (2021)[41]	Feasibility Study on Animal Models	Remotely-controlled robotic surgery system. Connection between operator station and the controlling terminal was achieved utilizing the 5G wireless network – Connected Surgeon to Surgical Robot	3 Swine Patient Models	Surgeries carried out successfully. No postoperative complications. Critical structures were easily identified and preserved. Delay recorded at 250ms.	-

Supplemental Table S1: Studies evaluating the IoT in Telesurgery or Surgical Telementoring

Huang et al (2021)[48]	Develoment of Prototype System	Augmented reality was utilized to superimpose preoperative 3D models of the target organ. The augmented reality overlay, was transmitted over the live feed of the camera, to a remote operator location – Connected Preoperative Imaging, Remote Surgeon and Surgical Robot	-	User-friendly system. Potential for reduction of surgical times. Potential for similar accuracy as conventional methods.	-
Sachdeva et al (2020) [49]	Develoment of Prototype Model	A novel approach for tracking the robotic tools in telesurgery, with the aid of a deep learning neural network that allowed for optical rather than sensory recognition – Connected Tracking Sensors to Data Collection Module	-	System was accurate in perceiving the positioning of the arms. Total latency time was 299 ms. Can further lessen latency times in telesurgery, to include areas with lower internet speeds.	System lacks depth perception.
Wirz et al (2015) [42]	Feasibility Study	Telesurgery approach for the removal of pituitary tumors over a distance of 800km – Connected Surgeon to Surgical Robot	-	No additional surgical times, low observed latency at 10,37ms.	Concerns over sterilization of the equipment required.

Supplemental Table S1: Studies evaluating the IoT in Telesurgery or Surgical Telementoring

Zheng et al (2020)[43]	Clinical Study	Integration of the 5G network on the telesurgical approach of laparoscopic surgical procedures. Distance between centers was at 3000 km – Connected Surgeon to Surgical Robot	4 Patients	All surgeries were completed successfully. No postoperative complications. Minimal blood loss was recorded. Latency of 264ms did not interfere with the procedures.	Need for further, larger studies on humans. Surgical systems need to be more compact to facilitate telesurgery. Gaps in medical laws need to be addressed.
Glenn et al (2016)[30]	Survey	A survey among rural surgeons, to determine views on telementoring, via high speed internet connections – Connected Mentors, Mentees and Presentation Tools	159 Surgeons	The vast majority of surgeons reported that they viewed telementoring as a positive addition to their surgical practice. High interest was recorded for live-feed intraoperative telementoring, as well as for trauma consultation.	Financial concerns were common among surgeons. Unavailability of reliable internet connection was raised as an issue. Lack of investments in specialized telementoring systems. Legal considerations.
Agrawal et al (2014)[9]	Study on Trainees	Several teaching methods were done in a distance learning fashion utilizing internet connection.– Connected Mentors, Mentees and Presentation Tools	38 Surgeons	90% of the participants found the implementation helpful in knowledge transmission.	Concerns over whether tele-consultation would be viable in low-resource settings.
Lenihan et al (2011)[31]	Clinical Study	Use of internet connection, in order for a distant surgeon to observe and proctor trainees on a	28 Patients	All surgeons found the proctoring extremely helpful in bettering their technique. Costs were avoided, as	Some problems with intraoperative audio transmission.

Supplemental Table S1: Studies evaluating the IoT in Telesurgery or Surgical Telementoring

		robotic surgery system – Connected Surgeon to Surgical Robot		live mentorship would have been expensive. No change in surgical outcomes.	
Park et al (2012)[44]	System Development	Development of a telesurgical robotic system. Incorporates connection of operator to robot, via the Internet – Connected Surgeon to Surgical Robot	-	The system setup allowed for feasibility in real-life use.	Some delays were recorded.
DeKastle (2009)[26]	Study on Trainees	Application of the broadcast of a live-feed surgical operation to nursing students, with simultaneous on-site teaching – Connected Mentors, Mentees and Presentation Tools	60 Students	The interactive lesson, was deemed influential for the choice of future career. Almost all of the trainees, found the live feed video helpful in their development.	Many students pointed out that this experience did not substitute being taught within the operating room itself.
Prince et al (2018)[14]	System Development	Development of a system for telementorship during live laparoscopic surgery.– Connected Mentors, Mentees and Presentation Tools	14 Trainees	The mentor could assume control of the trainee's system. Instrument movement of the mentor could be replayed for the mentee. The system can compare performances and provide feedback to the mentee. The trainees that used the system, scored higher on the	-

Supplemental Table S1: Studies evaluating the IoT in Telesurgery or Surgical Telementoring

				dexterity assessment than those who did not.	
Acemoglu et al (2020)[45]	Feasibility Study	Application of a 5G-based telesurgery system, with a robotic surgery interface.– Connected Surgeon to Surgical Robot	-	The surgeon reported confident control of all instruments. The operations were successful. Maximum delay was 140ms.	-
Tian et al (2020)[46]	Clinical Study	Application of a 5G-based telesurgery system, with a robotic surgery interface. – Connected Surgeon to Surgical Robot	12 Patients	All operations were carried out successfully. No postoperative complications were recorded. Accuracy was optimal.	Surgeons must be trained in robotic surgery. Telesurgery is not yet tried in complex and difficult cases.
Hinata et al (2014)[28]	Clinical Study	Comparison of telementoring versus live-mentoring in robotic surgery – Connected Mentors, Mentees and Presentation Tools	30 Patients	The telementoring system was reliable, with no interruptions of audio or video recorded. All postoperative measurements of the patients, were comparable	Requires reliable and fast internet connection.

Supplemental Table S1: Studies evaluating the IoT in Telesurgery or Surgical Telementoring

				between the two groups.	
Safier et al (2015)[20]	Study on Trainees	Evaluation of a remote monitoring and supervision system by surgical trainees – Connected Mentors, Mentees and Presentation Tools	21 Trainees	Significant positive feedback from residents. Trainees reported comfort with no negative impact on patient safety. All of them felt comfortable proceeding without live supervision.	-
Shin et al (2014)[34]	Study on Trainees	Comparison of live operationg room (OR) mentoring, with telementoring practices – Connected Mentors, Mentees and Presentation Tools	55 Patients	Mentors chose remote over on-site mentoring. Skill assessment scores were similar in the two groups.	17% of the wireless telementoring sessions were interrupted due to connection issues.
Din et al (2021)[27]	Clinical Study	Evaluation of a telementoring platform for corneal eye surgery. The platform utilized 5G network, to transmit video to a distant experienced surgeon– Connected Mentors, Mentees and Presentation Tools	3 Surgeons	Both the surgeons and the proctor evaluated the experience as extremely positive and helpful. No technical issues were recorded.	-

Supplemental Table S1: Studies evaluating the IoT in Telesurgery or Surgical Telementoring

Schalachta et al (2010)[21]	Clinical Study	Telementored laparoscopic surgery, with the use of the Internet for video and audio transmission – Connected Mentors, Mentees and Presentation Tools	20 Patients	The long-distance mentoring had a positive effect on surgical times.	-
Rojas-Munoz et al (2020)[16]	System Development	An AR telementoring system, where a distant experienced surgeon could view a live feed of an operating view, and in turn could provide instructions. – Connected Mentors, Mentees and Presentation Tools	-	First responders trained with the system, scored higher in surgical skills evaluationsand demonstrated safer techniques.	-
Liu et al (2021)[36]	System Development	An AR telementoring system, where a distant experienced surgeon could view a live feed of an operating view, and in turn could provide instructions. – Connected Mentors, Mentees and Presentation Tools	-	The trainee, could accurately replicate the mentor's hand motionsand succeeded in a mock procedure.	-

Supplemental Table S1: Studies evaluating the IoT in Telesurgery or Surgical Telementoring

Altieri et al (2020)[29]	Study on Trainees	Randomized Clinical Trial, comparing the live teaching of surgical energy devises use, with the corresponding telementored teaching – Connected Mentors, Mentees and Presentation Tools	65 Trainees	Post-course assessment was similar between the two groups. Long-term performance decline was non-inferior in the telementored group.	Costs of such events might be higher when telementoring is used.
Tel et al (2020)[66]	Study on Trainees	Surgical procedures were broadcast live for surgical trainees at a distance. The broadcast was projected on an interactive whiteboard, where participants could use their smart devices, to annotate during the surgery – Connected Mentors, Mentees and Presentation Tools	90 Trainees	Satisfaction index of 90% was achieved. No quality losses in image were noted.	
Rojas-Munoz et al (2020)[18]	Study on Trainees	An AR telementoring system, where a distant experienced surgeon could view a live feed of an operating view, and in turn could provide instructions. – Connected Mentors, Mentees	20 Trainees	The study group assigned to the telementoring system, achieved higher procedure-related scores, and were prone to less errors.	-

Supplemental Table S1: Studies evaluating the IoT in Telesurgery or Surgical Telementoring

		and Presentation Tools			
Rojas-Munoz et al (2020)[19]	Study on Trainees	An AR telementoring system, where a distant experienced surgeon could view a live feed of an operating view, and in turn could provide instructions. – Connected Mentors, Mentees and Presentation Tools	20 Trainees	The study group assigned to the telementoring system, achieved higher procedure-related scores, and were prone to less errors. participants with fewer experience, performed better when trained using the AR system.	The telementoring group, required more guidance time on average.
Lacy et al (2019)[37]	Clinical Study	The mentored surgical team was monitored with cameras and microphones, that transmitted data via a 5G network, The mentor also received live image feed of the laparoscope and provided live feedback and annotated guidance – Connected Mentors, Mentees, Smart Devices and Surgical Tools	2 Patients	The mentees rated with an almost perfect score, both the image transmission and the mentoring quality. Both surgeries were completed without complications.	Not enough data in order to systematically allow for pure telesurgical procedures. Surgeon experience is connected with the feasibility of the method.

Supplemental Table S1: Studies evaluating the IoT in Telesurgery or Surgical Telementoring

Netzer et al (2019)[38]	Study on Trainees	Comparison of telementoring support, with no support in BLS-instructed military medics – Connected Mentors, Mentees and Presentation Tools	33 Trainees	The telementored group, was significantly more efficient in diagnosing and treating pneumothorax.	Performance of thoracocentesis took longer in the telementored group.
Mitsuno et al (2019)[2]	Feasibility Study	An AR telementoring system, where a distant experienced surgeon could view a live feed of an operating view, and in turn could provide instructions. – Connected Mentors, Mentees and Presentation Tools	1 Trainee	Successful completion of operation. High satisfaction reported from trainer and trainee.	-
Andersen et al (2019)[10]	Study on Trainees	An AR telementoring system, where a distant experienced surgeon could view a live feed of an operating view, and in turn could provide instructions. – Connected Mentors, Mentees and Presentation Tools	10 Trainees	Participants in the telementoring group, achieved higher operative scores, and less surgical times.	Variability in patients, make pre-recorded operations less reliable.

Supplemental Table S1: Studies evaluating the IoT in Telesurgery or Surgical Telementoring

Kirkpatrick et al (2019)[35]	Study on Trainees	Comparison of telementoring support, with no support in BLS-instructed military medics – Connected Mentors, Mentees and Presentation Tools	7 Trainees	More people in the telementored group were able to perform the task. The mentors reported high satisfaction rates.	-
Rojas-Munoz et al (2019)[17]	Study on Trainees	Comparison of AR telementoring methods with simple audio and video telementoring – Connected Mentors, Mentees and Presentation Tools	20 Trainees	The AR system resulted in less errors, and less shifts from the target position.	Trainees with simple telementoring achieved less surgical times.
Dawe et al (2018)[25]	Study on Trainees	Evaluation of telementoring, in non-medical participants in performance of surgical tasks – Connected Mentors, Mentees and Presentation Tools	4 Trainees	All participants were successful in the tasks at hand. Participants felt that telementoring enhanced their capabilities.	-
Talbot et al (2018)[22]	Study on Trainees	An AR telementoring system, where a distant experienced surgeon could view a live feed of an operating view, and in turn could provide instructions. – Connected Mentors, Mentees	4 Trainees	Out of 16 simulated fasciotomies, 14 were successful.	Drops in bandwidth caused blurred videos at several instances.

Supplemental Table S1: Studies evaluating the IoT in Telesurgery or Surgical Telementoring

		and Presentation Tools			
Pia Poretta et al (2017)[64]	Clinical Study	Comparison of a remote telementoring protocol was evaluated with on-site mentoring – Connected Mentors, Mentees and Presentation Tools	49 Patients	No differences in postoperative outcomes.	Re-operation rates were lower when telementoring was not implemented.
Nguen et al (2018)[51]	Study on Trainees	Evaluation of a telementoring system that utilized audiovisual information, to assist in sleeve gastrectomy surgery – Connected Mentors, Mentees, Smart Devices and Surgical Tools	15 Trainees	The overall experience of the mentors and the mentees was rated at the highest level. All operations were successful	Scheduling limitations between mentors and mentees.
Moore et al (2017)[32]	Study on Trainees	After receiving training on operative techniques, novice surgeons submitted online videos of their operations in order to be assessed – Connected Mentors, Mentees and Presentation Tools	18 Trainees	Feasibility of asynchronous telementoring.	-

Supplemental Table S1: Studies evaluating the IoT in Telesurgery or Surgical Telementoring

Anderson et al (2017)[11]	Study on Trainees	An AR telementoring system, where a distant experienced surgeon could view a live feed of an operating view, and provide instructions by drawings that were superimposed on the operators' field of view. – Connected Mentors, Mentees and Presentation Tools	20 Trainees	Increased focus through the integration of mentors' commentary, and no need for attention shift.	High bandwidth required.
Snyderman et al (2016)[52]	Study on Trainees	Evaluation of a telementoring system that utilized audiovisual information, to assist in endoscopic endonasal surgery – Connected Mentors, Mentees and Presentation Tools	10 Patients	Adequate audio and video connection was maintained throughout. Mentees rated the experience with a perfect score.	-
Andersen et al (2016)[12]	Study on Trainees	An AR telementoring system, where a distant experienced surgeon could view a live feed of an operating view, and provide instructions by drawings that were superimposed on the operators' field of view. – Connected	20 Trainees	The AR system resulted in less errors, and less shifts from the target position.	Trainees with simple telementoring achieved less surgical times.

Supplemental Table S1: Studies evaluating the IoT in Telesurgery or Surgical Telementoring

		Mentors, Mentees and Presentation Tools			
Kirkpatrick et al (2015)[53]	Study on Trainees	Comparison of a remote telementoring protocol was evaluated with on-site mentoring – Connected Mentors, Mentees and Presentation Tools	12 Trainees	Mentees reported increased self-confidence.	Significantly more simulated blood loss, occurred with the remote telementoring group. Telementoring group, also utilized more surgical resources.
Fuertes-Guiro et al (2016)[1]	Clinical Study	Evaluation of a telementoring system that utilized audiovisual information, to assist in bariatric surgery – Connected Mentors, Mentees and Presentation Tools	20 Trainees	Telementoring of surgeons resulted in shorter hospital stays, shorter operative times and less complications.	-
Forgione et al (2015)[40]	Clinical Study	After receiving training on operative techniques, a novice surgeon performed 2 operations in a remote area, with telementoring assistance – Connected	2 Patients	Successful completion of both operations. The same surgeon continued to operate without the need for further guidance.	-

Supplemental Table S1: Studies evaluating the IoT in Telesurgery or Surgical Telementoring

		Mentors, Mentees and Presentation Tools			
Artsen et al (2021)[13]	Clinical Study	Evaluation of a telementoring system that utilized audiovisual information, to assist in gynecological surgery – Connected Mentors, Mentees and Presentation Tools	7 Patients	All participants reported high satisfaction with the application. Surgical times were shorter compared to live mentoring. No difference in complication rates.	-
Shabir et al (2021)[50]	System Development	Development of a telementoring system, which includes the superimposition of virtual laparoscopic tools controlled by the remote mentor– Connected Mentors, Mentees and Presentation Tools	-	The mentee was able to see and replicate the tool movements, without the mentor having to use actual surgical tools on a patient.	-
Greenberg et al (2021)[39]	Study on Trainees	At home surgical skills course. Utilization of a virtual mentoring platform. The mentor used live video feed and AR interface to annotate and guide the mentee – Connected Mentors, Mentees	14 Trainees	All participants were eventually able to participate in the course. Post-course evaluation showed a high satisfaction rate.	Most of the trainees reported logistic issues with the course. Cost of the course increased significantly. Coordination was an issue.

Supplemental Table S1: Studies evaluating the IoT in Telesurgery or Surgical Telementoring

		and Presentation Tools			
Trujilo Loli et al (2021)[23]	Study on Trainees	At home surgical skills course. Utilization of a video call platform – Connected Mentors, Mentees and Presentation Tools	40 Trainees	A notable increase in skill was noted after the course. Relatively low cost of the system.	-
Yanni et al (2020)[74]	System Development	Utilization of an IoT sensor network for intraoperative monitoring of the surgical patient.– Connected Mentors, Mentees and Monitoring Sensors	-	Intraoperative monitoring of basic patient parameters is expected to better the outcomes of telesurgery and telementoring Potential to include more sensors for other biological parameters.	-
