# Supplementary material for: A Smarter Health through the Internet of Surgical Things
Source: Sensors (Basel). 2022 Jun 17;22(12):4577. doi: 10.3390/s22124577 (PMC9231158; doi:10.3390/s22124577)
Supplement: Supplementary file 1 [file sensors-22-04577-s001.zip › sensors-1689180-supplementary/Supplemental Table S2.pdf]

Supplemental Table S2: Studies on Image-Guided Surgery incorporating IoT developments

**Studies Evaluating Image-Guided Surgery incorporating IoT developments**	**Type of Study**	Evaluated Method – Connected IoST entities	**No of patients studied**	**Proposed Advantages of IoST Image-Guided Surgery**	**Limitations of IoST Image-Guided Surgery**
Padilla et al (2015)[59]	In Silico Tool Development	Connected Preoperative Imaging and Intraoperative Surgical Tools	-	Interactive analysis of MRI data to assist in deep-brain stimulation. Identification of the placement of the microelectrode in real-time. Registration of 3D brain models.	Lack of large quantities of patient data
Ushimaru et al (2019)[5]	Feasibility study on animal models	Visualization of instrument use in laparoscopic surgery. – Connected Surgical Tools	5 Porcine Patient Models	All surgical instruments were tracked accurately.	-
Guo et al (2018)[8]	System Development	MRI device connected with a surgical robot, both connected to a central computer system - Connected Preoperative Imaging and Intraoperative Surgical Tools	-	Able to perform in confined space, tests on DBS electrode insertion show optimal accuracy, direct visualization, telesurgery, saves on operating times, as well as on nursing costs	Lack of clinical trials for evaluation on humans.
Li et al. (2015)[54]	System Development	MRI-guided robotic system. MRI device connected with a surgical robot - Connected Preoperative Imaging and Intraoperative Surgical Tools	-	Interactive updates of the anatomy, accountability for intracranial structure shift. High accuracy, time efficient when compared to CT-MRI coupling	Lack of clinical trials for evaluation on humans.

Supplemental Table S2: Studies on Image-Guided Surgery incorporating IoT developments

Luis et al (2021)[12]	Clinical Study	Extended reality (XR)system, that provided VR preoperative planning, and AR intraoperative assistance– Connected Pre- and Intra-operative Imaging	49 Patients	Aided in the identification of vital structures, and their preservation. Real time functions, improved spatial awareness	No comparison group included in the study. No measures of accuracy or surgical times.
Ivan et al (2021)[56]	Clinical Study	Augmented Reality glasses, that incorporated preoperative MRI imaging,. Open Site is used for the generation of a holographic surface through importing of the MRI imaging from an online cloud service – Connected Pre- and Intra-operative Imaging	11 Patients	Accuracy improved with experience. AR provided real time information on the 3D imaging of the tumors. Wireless application, not overly costly.	Not significantly more accurate than traditional navigation. The value of AR guidance needs to be evaluated in larger studies.
Fan et al (2016)[62]	Clinical Study	Updated MR imaging generation intraoperatively, by utilizing intraoperative stereovision images – Connected Pre- and Intra-operative Imaging	20 Patients	Improved accuracy and alignment, effective decompensation for brain deformation during surgery	-

Supplemental Table S2: Studies on Image-Guided Surgery incorporating IoT developments

Kiarostami et al (2020)[58]	Pre-Clinical study	CT scans were imported in an AR generating HoloLens. – Connected Pre- and Intra-operative Imaging	30 simulated attempts	Accuracy significantly increased for the more inexperienced surgeon.	No significant increase of accuracy when compared to traditional surgery, in the experienced surgeon.
Hu et al (2013) [65]	System Development	AR - assisted image guided endocranial surgery. The live video was incorporated with preoperative scans,. Includes a live-tracked marker. – Connected Pre- and Intra-operative Imaging	-	Easy model-to-patient registration. Inexpensive equipment. Expected to be suitable for real-life use.	Use of a real-life tracker was necessary. Instances of manual recalibration were noted, that can cause reduction of the system's accuracy.
Momi et al (2015)[57]	Clinical Study	Integration of a neuronavigator system, with a 3D model creation application, intraoperatively– Connected Pre- and Intra-operative Imaging	12 Patients	Accurate assessment of intraoperative brain shift.	Anatomical landmarks must be chosen carefully in order to not interfere with the procedure.

Supplemental Table S2: Studies on Image-Guided Surgery incorporating IoT developments

Luo et al (2016)[73]	System Development	A system for liver surgery planning. Incorporates preoperative CT imaging and CT angiography. Utilizes 3 interconnected server systems.– Connected Pre- and Intra-operative Imaging	-	Successful creation of 3D model, including intrahepatic vessels. Expected to provide guidance in hepatectomy.	-
Yoon et al (2017)[61]	Clinical Study	. Preoperative imaging data was incorporated with an intraoperative guidance system and a video capture system. The integrated images were then projected through a wireless cloud system. – Connected Pre- and Intra-operative Imaging	2	No complications were recorded. Limited obstruction of surgical field view. Voice-controlled system. Confirmation of positioning and tracking of the catheter trajectory in real time.	Short battery life of the system. Size of display can be tricky. Method requires validation in larger studies.
Eftekhar (2016)[63]	Clinical Study	Incorporation of a mobile app, within IGS for neurosurgery. Preoperative imaging can be downloaded within the application. Overlapping of preoperative image on the camera feed– Connected Pre- and Intra-operative Imaging	11	Availability of smart phones. Application is free of charge. No need for head fixation. No requirement of training.	Anatomic landmarks to be used are often difficult to appreciate. Manual adjustment of the overlay. Limited number of patients.

Supplemental Table S2: Studies on Image-Guided Surgery incorporating IoT developments

Watanabe et al (2016)[60]	Clinical Study	Preoperative imaging utilized to render 3D models for the operation. Images were Intraoperatively overlaid in the live feed of a tablet's, – Connected Pre- and Intra-operative Imaging	6	Volumetric, three dimensional navigation was possible. No need to alter the surgeon's gaze between field and screen. No blind spots in navigation.	System does not compensate for brain shifts during surgery. A lag of 0,4 seconds between the physical movements of the tablet, and the corresponding image change was recorded.
