# Supplementary material for: A Smarter Health through the Internet of Surgical Things
Source: Sensors (Basel). 2022 Jun 17;22(12):4577. doi: 10.3390/s22124577 (PMC9231158; doi:10.3390/s22124577)
Supplement: Supplementary file 1 [file sensors-22-04577-s001.zip › sensors-1689180-supplementary/Supplemental Table S3.pdf]

Supplemental Table S3: Studies evaluating the IoT in Surgical Patient Telemonitoring

Studies Evaluating the IoT in Surgical Patient Telemonitoring	Type of Study	Evaluated Approaches – Connected IoST entities	No of Participants	Proposed Advantages	Limitations
Omboni et al (2019)[7]	Clinical Study	An internet-based telemedicine platform that allowed for the recording of blood pressure, laboratory results, anthropometric data, as well as personal medical history. A portable oscillometer device was coupled with the platform. – Connected Monitoring Sensors to Distant Physician	1200 Patients	Most patients remained consistent with the study requirements. The in-office visits were reduced. The vast majority of patients were able to use the platform efficiently.	The requirement for additional oscillometer use brought about some technical difficulties.
Wildemeersch et al (2018)[66]	Clinical Study	An enhanced recovery programme after chest wall surgery, based around patient monitoring and education– Connected Monitoring Sensors to Distant Physician	29 Patients	Telemonitoring at home was achieved at the vast majority of patients.	Some adherence difficulties on behalf of the patients were noted.
Msayib et al (2017)[67]	System Development	A postoperative, at-home monitoring system for knee arthroplasty patients.– Connected Monitoring Sensors to Distant Physician	-	The system is expected to simplify postoperative monitoring, enable telemonitoring and automate the	-

Supplemental Table S3: Studies evaluating the IoT in Surgical Patient Telemonitoring

				postoperative period.	
Paysson et al (2015)[68]	Clinical Study	An Internet based system, for transmission of home-spirometry measurements for patients post lung transplantation . – Connected Monitoring Sensors to Distant Physician	59 Patients	Agreement of spirometry values was satisfactory, between in-hospital and home measurements.	Patient adherence proved to be an issue.
Jonker et al (2020)[69]	Clinical Study	Evaluation of a telemonitoring system for postoperative patients that underwent cancer-related surgery. Participants utilized a mobile application connected to several smart devices that recorded postoperative parameters . – Connected Monitoring Sensors to Distant Physician	65 Patients	Within the participants, postoperative data were collected with ease.	Such methods are not available for the technologically illiterate, those without internet access and elderly patients with high mental burden.
Cos et al (2021)[3]	Clinical Study	A wearable device connected to the Internet was used for the preoperative evaluation of patients undergoing pancreatectomy. – Connected	54 Patients	The collected data could accurately predict postoperative outcomes when used for model creation. Patient	It is possible that elderly patients will be left out of such initiatives.

Supplemental Table S3: Studies evaluating the IoT in Surgical Patient Telemonitoring

		Monitoring Sensors to Distant Physician		adherence was excellent.	
Cornelis et al (2021)[70]	Clinical Study	A home-based exercise programme delivered through the Internet with the aid of wearable devices,. – Connected Monitoring Sensors to Distant Physician	20 Patients	All of the patients adhered to the programme. Near-perfect satisfaction scores. All claudication-related parameters were improved.	Need for Internet access.
Zetterman et al (2011) [71]	Clinical Study	Preoperative evaluation of patients in a virtual environment . – Connected Monitoring Sensors to Distant Physician	No of patients inaccessible	The majority of patients preferred the virtual to live evaluation. No technical difficulties were recorded.	About 1 in 10 patients would still prefer a live preoperative assessment.
Mullen-Fortino et al (2019)[71]	Clinical Study	Comparison of preoperative assessment in the form of teleconsulting, with live assessment. – Connected Monitoring Sensors to Distant Physician	361 Patients	Teleconsulting saved time for patients and providers alike. All of the patients were happy with their evaluation. There were no appointment cancellations in the tele-evaluation group.	-
Kim et al (2018)[6]	Clinical Study	A Doppler cuff was implanted distal to blood vessel anastomoses, for the creation of skin flaps.– Connected Monitoring Sensors to Distant Physician	18 Patients	Real-time monitoring of the skin flaps, allowed for greater survivability rates of the grafts. Real time monitoring	-

Supplemental Table S3: Studies evaluating the IoT in Surgical Patient Telemonitoring

				was available everywhere.	
Vilallonga et al (2013)[72]	Clinical Study	Comparison of at-home follow-up, using the Internet, with standard follow-up after bariatric surgery. – Connected Monitoring Sensors to Distant Physician	10 Patients	More than 90% of the patients reported high satisfaction and positively evaluated timesaving.	Lack of Internet access and Computer knowledge is limiting availability.
Caggianese et al (2017)[75]	System Development	A rehabilitation system for cardiac surgery patients. Input consists of motion tracking and biomedical sensors attached to the patient.– Connected Monitoring Sensors to Distant Physician	-	Long distance patient monitoring. Creation of a patient data repository utilization of biomedical signals.	-
Wang et al (2020)[76]	Clinical Study	Comparison of wireless, intelligent system for postoperative PCA with traditional PCA. – Connected Monitoring Sensors to Distant Physician	6.191 Patients	Moderate or Severe pain was lower with the Wi-PCA group. Postoperative nausea was also reduced. Coupling the system with biosensors has the potential to create an A.I.-controlled analgesia delivery system.	Major safety concerns regarding the nature of analgesia drugs and their management by an A.I.

Supplemental Table S3: Studies evaluating the IoT in Surgical Patient Telemonitoring

McGillion et al (2020)[77]	Clinical Study	Utilization of a biosensor-based wireless system for long-range postoperative patient monitoring. – Connected Monitoring Sensors to Distant Physician	11 Patients	Both trained nurses and patients would highly recommend the system after the trial period. All patients managed to connect their devices and obtain regular vital sign readings.	Some patients experienced difficulties with using the system. Practice was needed prior to engagement.
Rouholiman et al (2018)[81]	System Development	Use of an ostomy alert sensor that could measure ostomy output connected wirelessly to a smartphone user interface. – Connected Monitoring Sensors to Distant Physician	20 Patients	Anticipated to improve quality of life, infection rates and reduce inpatient visits.	-
Colomina et al (2021)[78]	Clinical Study	Use of an Internet-based preoperative monitoring platform coupled with an activity-tracking sensor. – Connected Monitoring Sensors to Distant Physician	29 Patients	Patients in the study group had 50% lower unplanned visits to their physician.	Need for patients to be technologically adept.
Holmes et al (2020)[79]	Clinical Study	Assessment of movement, location, and posture sensor data (transmitted over the Internet) from postoperative– Connected	3 Patients	The system could identify trends in sleep quality, movement and mobility of the patient.	Need for simplified data visualization for healthcare professionals.

Supplemental Table S3: Studies evaluating the IoT in Surgical Patient Telemonitoring

		Monitoring Sensors to Distant Physician			
Kong et al (2021)[80]	Clinical Study	Remote postoperative patient monitoring, using portable wireless sensors– Connected Monitoring Sensors to Distant Physician	24 Patients	The system was able to detect postoperative complications efficiently. Medical staff responsible for system handling, reported excellent satisfaction scores.	-
Ohta et al (2015)[4]	System Development	Development of an NFC-based wireless sensor that can be swallowed and magnetically anchored to the gastric wall. Wireless data transmission is continuous. – Connected Monitoring Sensors to Distant Physician		Avoidance of surgical procedure in order to implant an indwelling sensor. The sensors were able to successfully record ECG, heart rate and gastric pH in a preliminary study. Avoidance of the inconvenience used by the wearable devices.	
