# Supplementary figures and images for: A Smarter Health through the Internet of Surgical Things
Source: Sensors (Basel). 2022 Jun 17;22(12):4577. doi: 10.3390/s22124577 (PMC9231158; doi:10.3390/s22124577)

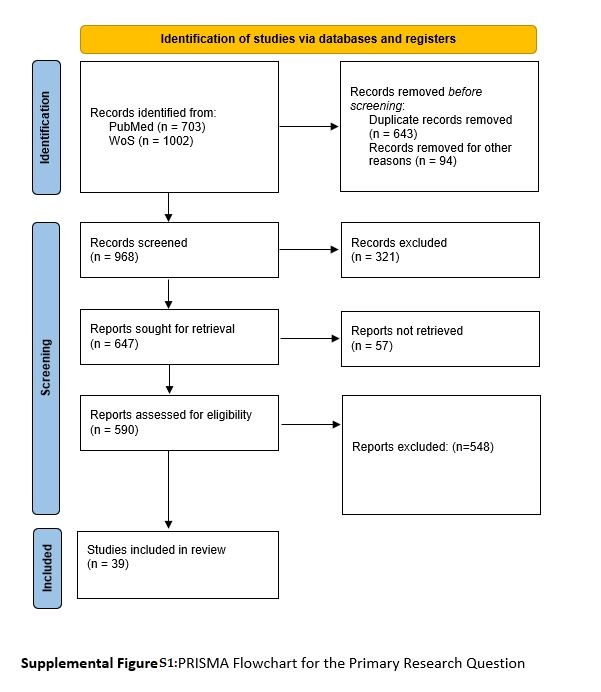

Supplement: Supplementary file 1 [file sensors-22-04577-s001.zip › sensors-1689180-supplementary/Supplemental Figure S1.JPG]

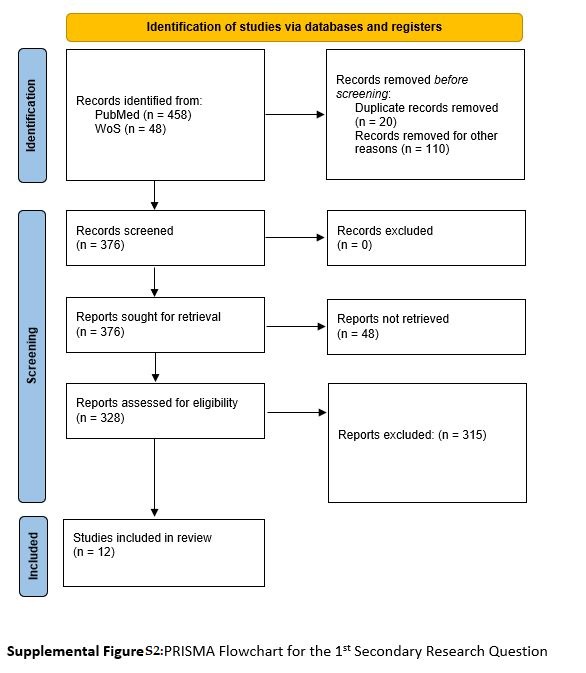

Supplement: Supplementary file 1 [file sensors-22-04577-s001.zip › sensors-1689180-supplementary/Supplemental Figure S2.JPG]

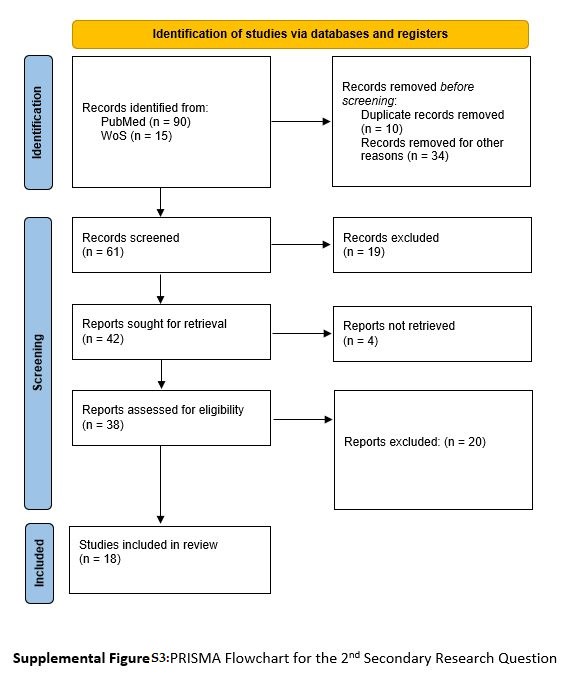

Supplement: Supplementary file 1 [file sensors-22-04577-s001.zip › sensors-1689180-supplementary/Supplemental Figure S3.JPG]

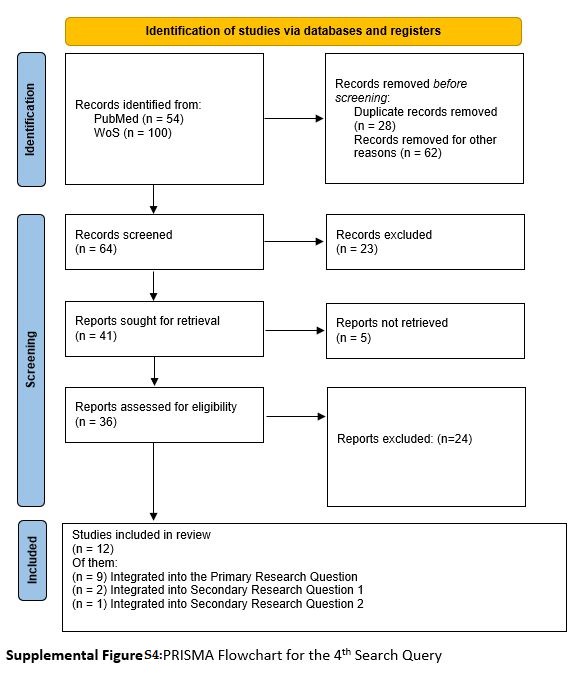

Supplement: Supplementary file 1 [file sensors-22-04577-s001.zip › sensors-1689180-supplementary/Supplemental Figure S4.JPG]
